# High-Dose Polymerized Hemoglobin Fails to Alleviate Cardiac Ischemia/Reperfusion Injury due to Induction of Oxidative Damage in Coronary Artery

**DOI:** 10.1155/2015/125106

**Published:** 2015-06-16

**Authors:** Qian Yang, Wei Wu, Qian Li, Chan Chen, Ronghua Zhou, Yanhua Qiu, Ming Luo, Zhaoxia Tan, Shen Li, Gang Chen, Wentao Zhou, Jiaxin Liu, Chengmin Yang, Jin Liu, Tao Li

**Affiliations:** ^1^Department of Anesthesiology and Translational Neuroscience Center, West China Hospital, Sichuan University, Chengdu 610041, China; ^2^Department of Medicinal Chemistry, School of Pharmacy, Chengdu Medical College, Chengdu 610083, China; ^3^Department of Anesthesiology, Chengdu Military General Hospital, Chengdu 610083, China; ^4^Institute of Blood Transfusion, Chinese Academy of Medical Sciences, Chengdu 610052, China

## Abstract

*Objective*. Ischemia/reperfusion (I/R) injury is an unavoidable event for patients in cardiac surgery under cardiopulmonary bypass (CPB). This study was designed to investigate whether glutaraldehyde-polymerized human placenta hemoglobin (PolyPHb), a hemoglobin-based oxygen carrier (HBOC), can protect heart against CPB-induced I/R injury or not and to elucidate the underlying mechanism. *Methods and Results*. A standard dog CPB model with 2-hour cardiac arrest and 2-hour reperfusion was established. The results demonstrated that a low-dose PolyPHb (0.1%, w/v) provided a significant protection on the I/R heart, whereas the high-dose PolyPHb (3%, w/v) did not exhibit cardioprotective effect, as evidenced by the impaired cardiac function, decreased myocardial oxygen utilization, and elevated enzymes release and pathological changes. Further study indicated that exposure of isolated coronary arteries or human umbilical vein endothelial cells (HUVECs) to a high-dose PolyPHb caused impaired endothelium-dependent relaxation, which was companied with increased reactive oxygen species (ROS) production, reduced superoxide dismutase (SOD) activity, and elevated malonaldehyde (MDA) formation. Consistent with the increased oxidative stress, the NAD(P)H oxidase activity and subunits expression, including gp91^phox^, p47^phox^, p67^phox^, and Nox1, were greatly upregulated. *Conclusion*. The high-dose PolyPHb fails to protect heart from CPB-induced I/R injury, which was due to overproduction of NAD(P)H oxidase-induced ROS and resultant endothelial dysfunction.

## 1. Introduction

Ischemia/reperfusion (I/R) injury is harmful to cardiovascular system and responsible to cardiac infarction, which is thought to be involved in the severity and outcome of ischemic heart disease [1]. For these patients, percutaneous intervention or surgical procedure under cardiopulmonary bypass (CPB) is usually adopted to achieve coronary artery revascularization, but revascularization and cardiac arrest during CPB may induce I/R injury in myocardium [2]. Therefore, I/R injury is the major cause of death and poor prognosis of patients in cardiac surgery and transplantation.

Hemoglobin-based oxygen carriers (HBOCs) are red blood cell substitutes under development for more than three decades [3]. Our previous work and other studies indicated that HBOCs are promising candidates to prevent many organs from I/R injury [4–7]. Functionally, they allow delivery of more oxygen (O_2_) to hypoxic tissues due to their higher O_2_ affinity, lower viscosity, and smaller mean diameter than human erythrocytes. Mechanistic studies suggested that this effect is related to attenuation of myocardial apoptosis, oxidative stress, and nitroso-redox imbalance [8, 9]. However, this protection was not observed in clinical settings. A meta-analysis by Natanson et al. [10] demonstrated that those patients receiving a HBOC have a statistically increased risk of death and myocardial infarction. To address this discrepancy, this study employed a more clinically relevant animal model—dog CPB model—to investigate the effect of glutaraldehyde-polymerized human placenta hemoglobin (PolyPHb) with different dosage on cardiac I/R injury.

## 2. Materials and Methods

All animal experimental procedures were performed in accordance with the policies of the Animal Care and Use Committee of Sichuan University and conformed to the* Guide for the Care and Use of Laboratory Animals* published by the US National Institutes of Health (NIH Publication Number 85-23, revised 1996).

### 2.1. Preparation of Hemoglobin-Based Oxygen Carrier

PolyPHb, a HBOC developed in China, was prepared as reported previously with some modifications [11]. Briefly, purified and viral inactivated fresh human placenta hemoglobin was modified with bis(3,5-dibromosalicyl) fumarate to achieve optimal O_2_ affinity. After cross-linkage with glutaraldehyde, the mixture was subjected to ultrafiltration and molecular sieve chromatography. Before being used, the PolyPHb was mixed with St. Thomas' solution (STS) to a final concentration of 0.1 gHb/dL or 3 gHb/dL and then equilibrated with 95% O_2_ and 5% CO_2_ at 37°C for 15 min.

### 2.2. Dog Cardiopulmonary Bypass Model

A beagle dog cardiopulmonary bypass (CPB) model was established as described previously [12]. In brief, adult male beagle dogs, weighing 8–10 kg, were used. After induction (4 mg/kg propofol, 0.1 mg/kg midazolam, and 5 *μ*g/kg fentanyl) and muscle relaxation (1 mg/kg scoline), all the dogs were intubated with an Fr. 7.5 endotracheal tube and mechanically ventilated using an air/O_2_ mixture (1 : 4) with tidal volume 10 mL/kg (Datex-Ohmeda Excel 210, Soma Technology, Cheshire, Connecticut, USA). Each group received a continuous infusion of fentanyl at 0.3 *μ*g/kg/min and vecuronium bromide at 0.2 mg/kg/hr during surgery. Anesthesia was maintained with 150 *μ*g/kg/min propofol. After heart exposure through a mid-sternal incision and heparinization (3 mg/kg), the ascending aorta and the right atrial appendage were cannulated. The CPB circuit was composed of a rolling pump (StÖckert II, Munich, Germany), a membrane oxygenator (1500 mL/min, Kewei Medical Ltd., Guangdong, China), and an arterial filter (Kewei Medical Ltd.). The CPB was primed with Lactate Ringer's solution containing 5% sodium bicarbonate (10 mL/L), 20% mannitol (2.5 mL/L), furosemide (0.5–1.0 mg/L), dexamethasone (5 mg/L), heparin (10 mg/L), and 10% potassium chloride (5 mL/L). Also, a 10% calcium gluconate (2–4 mL) was added every 30 min for 4 times.

### 2.3. Experimental Protocol

**Figure 1:**
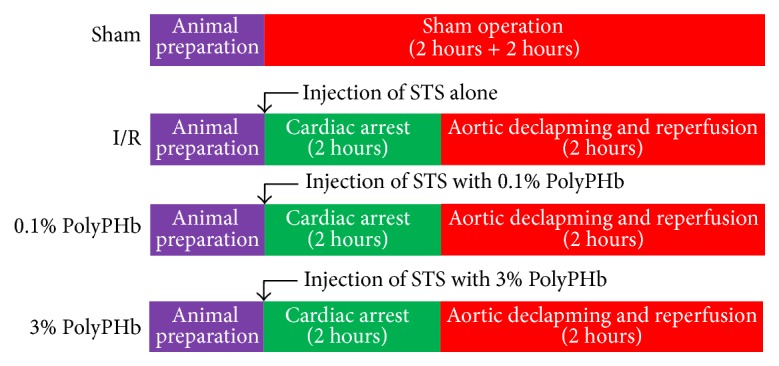
A schematic representation of the experimental protocol. After respective treatment, the hearts were subjected to 2-hour cardiac arrest and were reperfused for 2 hours. PolyPHb: glutaraldehyde-polymerized human placenta hemoglobin; STS: St. Thomas' solution.

The experimental protocol is schematically illustrated in Figure 1. Twenty adult male beagle dogs were divided into 4 groups (*n* = 5): Sham group, I/R group, 0.1% PolyPHb group, and 3% PolyPHb group. Except for the Sham group, hearts in other 3 groups were arrested by intra-aortic infusion of 40 mL/kg STS alone (I/R group), STS with 0.1 gHb/dL PolyPHb (0.1% PolyPHb group), or STS with 3 gHb/dL PolyPHb (3% PolyPHb group). After 2 hours of cardiac arrest, the hearts were reperfused for 2 hours by aortic declamping. The hearts without cardiac arrest and reperfusion were allocated into the Sham group. After the experiment, all the dogs were sacrificed with an intravenous bolus injection of sodium pentobarbital (120 mg/kg).

### 2.4. Measurement of Hemodynamic Parameters

A water-filled latex balloon attached to a pressure sensor (model SP844; MEMSCAP Inc., Durham, NC) was inserted into the left ventricle (LV) via the mitral valve. Then, the cardiac functional parameters including heart rate (HR), LV systolic pressure (LVSP), and LV end-diastolic pressure (LVEDP) were collected by a PowerLab data-acquisition system (ADInstruments Pty, Bella Vista, NSW, Australia). Also, a Swan-Ganz Float Catheter (Number 7, Edwards Laboratories, Irvine, CA, USA) was inserted via femoral vein and advanced to pulmonary artery to measure cardiac output (CO), pulmonary artery wedge pressure (PAWP), pulmonary arterial pressure (PAP), central venous pressure (CVP). Mean arterial pressure (MAP) was monitored by a polyethylene catheter placed in the left femoral artery.

### 2.5. Calculation of Cardiac O_2_ Utilization

Blood samples from artery and coronary vein sinus were collected. To assess the level of cardiac O_2_ utilization, cardiac O_2_ consumption (VO_2_) and O_2_ extraction index (O_2_EI) were calculated from the values of CO, hemoglobin concentration (Hb), arterial O_2_ partial pressure (PaO_2_), venous O_2_ partial pressure (PvO_2_), arterial O_2_ saturation (SaO_2_), and venous O_2_ saturation (SvO_2_) (ABL800 FLEX blood gas analyzer, Radiometer Medical A/S, Copenhagen, Denmark) by using following formula:

(1) VO2mL/min⁡=CO×1.38×Hb×SaO2−SvO2           +0.0031×(PaO2−PvO2)×10

(2) O2EI%=1−1.38×Hb×SvO2+0.0031×PvO21.38×Hb×SaO2+0.0031×PaO2      ×100%.

### 2.6. Determination of Myocardial Enzyme Release

Myocardial necrosis estimated by the releases of creatine kinase-MB (CK-MB), lactate dehydrogenase (LDH), and cardiac troponin-I (cTnI) in plasma were measured as described previously [8].

### 2.7. Measurement of Vascular Reactivity on Isolated Vessel Rings

Arterial rings (3-4 mm in length) from beagle dog coronary artery, free of fat and connective tissue, were mounted between two stainless steel hooks in organ bath chambers (PanLab Systems, Harvard apparatus, Barcelona, Spain). Each chamber contained 10 mL of Krebs-Henseleit (KH) solution (118 mM NaCl, 4.7 mM KCl, 1.2 mM KH_2_PO_4_, 1.2 mM MgSO_4_, 1.77 mM CaCl_2_, 25 mM NaHCO_3_, 11.4 mM glucose; pH 7.4, 37°C) and aerated continuously with 95% O_2_ and 5% CO_2_. Special attention was paid during the preparation to avoid damaging endothelium. During 60 minutes of equilibration period, resting tension of 3.5 g was periodically adjusted and the KH solution was changed every 30 minutes. The arterial viability was checked by stable and reproducible constriction to the addition of potassium chloride (KCl, 60 mM). Contracted arteries were then washed and subjected to 30 minutes of equilibration. After that, these arteries were incubated with KH buffer alone (Control group), KH buffer with 0.1% PolyPHb or 3% PolyPHb for 10 minutes and isometric tension of each vessel was recorded. To measure the effect of HBOC on endothelium, isolated coronary arterial rings were incubated with PolyPHb at 37°C for 2 hours. After washed and equilibrated for 60 minutes under resting tension of 3.5 g, these arteries were evoked using phenylephrine (10^−7^ M) to elicit reproducible contractile responses. Acetylcholine (ACh; 1 × 10^−8^ to 1 × 10^−4^ M) or sodium nitroprusside (SNP; 1 × 10^−10^ to 1 × 10^−6^ M) was then progressively added to induce endothelium-dependent or -independent relaxation, respectively.

### 2.8. Oxidative Stress and NAD(P)H Oxidase Activity Assays

Isolated dog coronary artery after treatment was embedded in aluminium cups of about 1 mL of OCT resin (Tissue Tek, Sakura, USA) and frozen in liquid nitrogen. To assess reactive oxygen species (ROS) production, cryosections (8 *μ*m) were stained with the superoxide-sensitive dye dihydroethidine (DHE, 10 *μ*M in PBS) and incubated for 30 minutes at 37°C. Red DHE fluorescence was detected with Olympus BX51 microscope and DP70 digital camera (Olympus corp.) at room temperature. Also, human umbilical vein endothelial cells (HUVECs) after treatment were incubated with DHE (10 *μ*M) for 30 minutes; then ROS production was quantified by fluorescent measurement under Em/Ex = 480/580 nm (LS55 fluorescence spectrometer, Perkin-Elmer corp., Boston, MA, USA). As markers of oxidative stress, the superoxide dismutase (SOD) activity and malondialdehyde (MDA) formation in HUVECs were also measured by using commercially available kits (Nanjing Jiancheng corp., Nanjing, China). The NAD(P)H oxidase activity of HUVECs was measured as described previously [13]. Briefly, 20 *μ*g of protein was incubated with DHE (10 *μ*M) and DNA (1.25 *μ*g/mL) in PBS with the addition of NAD(P)H (50 *μ*M), in a final volume of 120 *μ*L, for 30 minutes at 37°C in the dark. Fluorescence intensity was recorded in a microplate reader under Em/Ex = 480/580 nm (LS55 fluorescence spectrometer).

### 2.9. Immunohistochemistry

Paraffin sections (5 *μ*m) or cryosections (8 *μ*m) of dog coronary arteries were prepared and stained for P46^phox^, P67^phox^, gp91^phox^, Nox1, Nox4, and von Willebrand factor (vWF) by using standard and widely accepted immunostaining techniques. The vWF was used to indicate endothelium. Moreover, paraffin sections of dog LV tissue were stained with hematoxylin and eosin (H&E) and assessed in a blinded fashion by a pathologist for the following histological examination: hyaline change, cloudy swelling, fatty change, inflammatory infiltration, perinuclear halo, interstitial edema, and acute myocardial necrosis. Semiquantitative analysis of histopathological changes was performed using an arbitrary grading system from score 0 to 5 (score 0: <10% positive cells; score 1: 10%–20% positive cells; score 2: 21–30% positive cells; score 3: 31–40% positive cells; score 4: 41–50% positive cells; score 5: >50% positive cells).

### 2.10. Statistical Analysis

All values are presented as mean ± SD. An unpaired Student's *t*-test was used to detect significant differences when two groups were compared. One-way or two-way ANOVA was used to compare the differences among three or more groups followed by Bonferroni's multiple comparison tests as applicable (SPSS 16.0 software).* P* values < 0.05 were considered statistically significant.

## 3. Results

### 3.1. High-Dose PolyPHb Failed to Improve Cardiac Function after CPB-Induced I/R Injury

**Figure 2:**
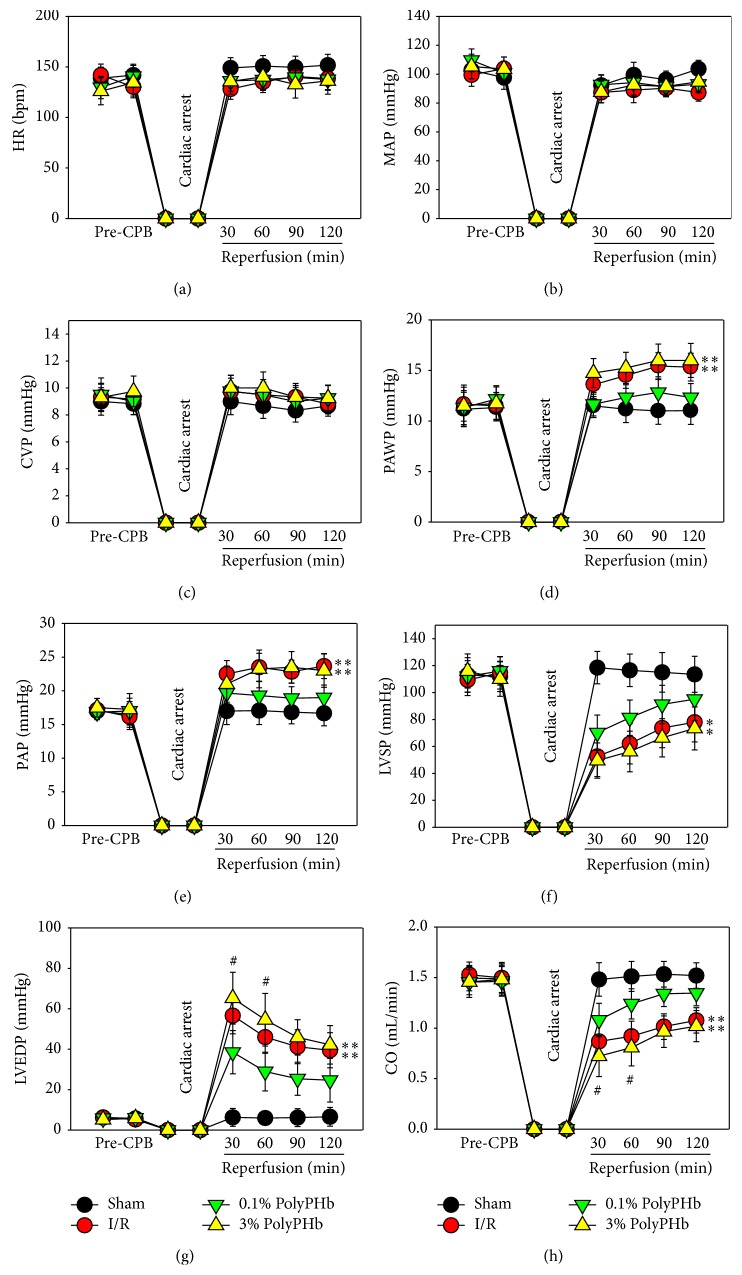
The HR (a), MAP (b), CVP (c), PAWP (d), PAP (e), LVSP (f), LVEDP (g), and CO (h) at baseline and during 2-hour of reperfusion (*n* = 5). Values are presented as mean ± SD. ^*^ *P* < 0.05 and ^**^ *P* < 0.01 versus the 0.1% group; ^#^ *P* < 0.05 versus the I/R group. HR: heart rate; MAP: mean arterial pressure; CVP: central venous pressure; PAWP: pulmonary artery wedge pressure; PAP: pulmonary arterial pressure; LVSP: left ventricular systolic pressure; LVEDP: left ventricular end-diastolic pressure; CO: cardiac output.

**Figure 3:**
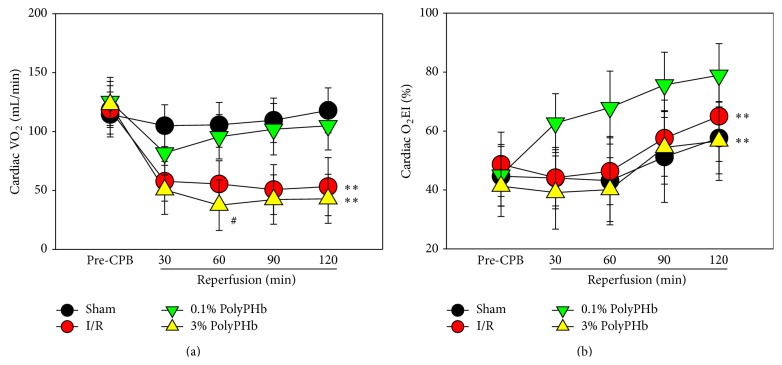
The cardiac utilization, including VO_2_ (a) and O_2_EI (b) at baseline and during 2 hours of reperfusion (*n* = 5). Values are presented as mean ± SD. ^**^ *P* < 0.01 versus the 0.1% group; ^#^ *P* < 0.05 versus the I/R group. VO_2_: cardiac oxygen consumption; O_2_EI: oxygen extraction index.

All the hemodynamic parameters at baseline, as well as the HR, CVP, and MAP during reperfusion, were not different among groups (Figures 2(a)–2(c)). Treatment with the low-dose PolyPHb exhibited cardioprotective effect. Increase of the dosage of PolyPHb did not enhance this effect, as shown by the increased PAWP, PAP, and LVEDP and reduced LVSP and CO (all *P* < 0.001 versus the Sham group; Figures 2(d)–2(h)). The recovery of LVEDP and CO during the first 60 minutes of reperfusion were even worse as compared to the I/R group (*P* < 0.05 and *P* < 0.05, respectively; Figures 2(g) and 2(h)). In addition, the 0.1% PolyPHb alleviated the reduction of cardiac VO_2_ and elevated O_2_EI as compared to the I/R group, while the 3% PolyPHb failed to improve these parameters and further decreased cardiac VO_2_ at 60 minutes of reperfusion (*P* < 0.05 versus the I/R group; Figure 3).

### 3.2. High-Dose PolyPHb Did Not Reverse Myocardial Necrosis after I/R Injury

**Figure 4:**
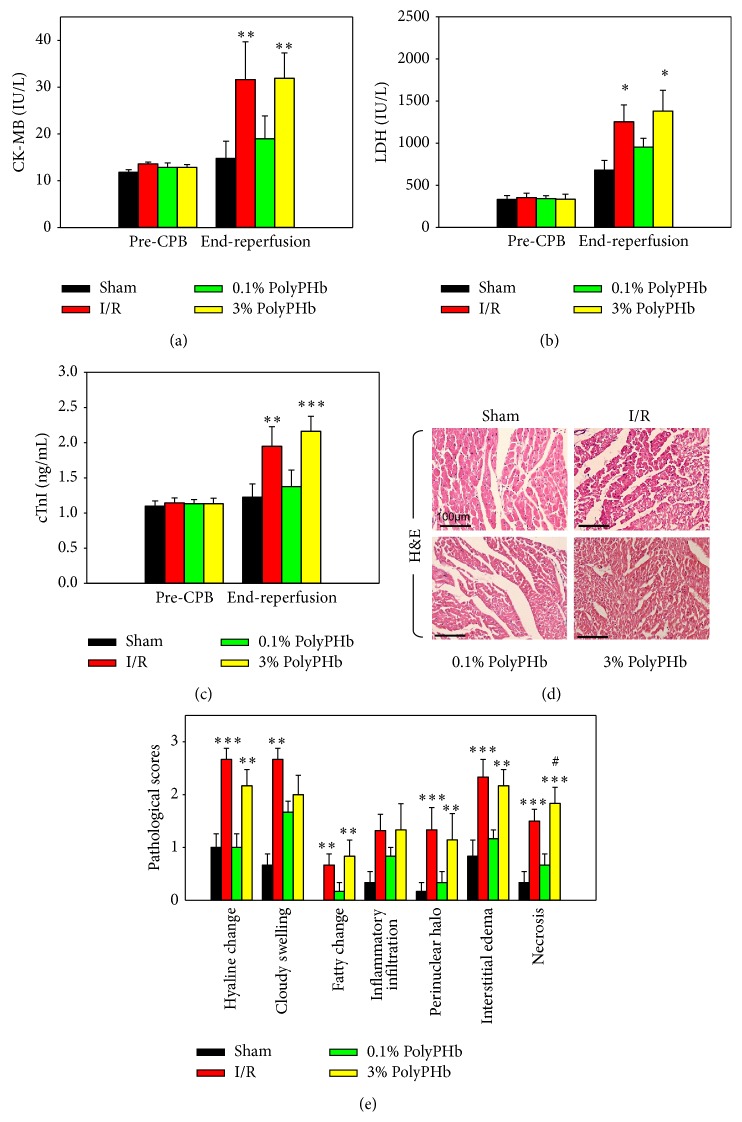
Total CK-MB (a), LDH (b), and cTnI (c) releases at baseline and after 2-hour reperfusion (*n* = 6). (d) Representative photomicrographs of H&E-stained left ventricular tissue section (*n* = 5). Scale bar: 100 *μ*m. (e) Pathological scores for hyaline change, cloudy swelling, fatty change, inflammatory infiltration, perinuclear halo, interstitial edema, and acute myocardial necrosis. Values are presented as mean ± SD. ^*^ *P* < 0.05, ^**^ *P* < 0.01, and ^***^ *P* < 0.001 versus the 0.1% PolyPHb group; ^#^ *P* < 0.05 versus the I/R group. CK-MB: creatine kinase-MB; LDH: lactate dehydrogenase; cTnI: cardiac troponin-I; H&E: hematoxylin and eosin.

As markers of myocardial necrosis, the levels of CK-MB, LDH, and cTnI in plasma were greatly increased in the I/R group. Less cardiac enzymes release was observed in the 0.1% PolyPHb group, whereas in the 3% PolyPHb group, the enzymes release was still in a high level and not different from the I/R group (Figures 4(a)–4(c)). Moreover, the results of H&E staining showed that the 3% PolyPHb did not limit myocardial histopathological changes after I/R injury and further increased myocardial necrosis (*P* < 0.05 versus the I/R group; Figures 4(d) and 4(e)).

### 3.3. High-Dose PolyPHb Impaired Endothelium-Dependent Vasorelaxation

**Figure 5:**
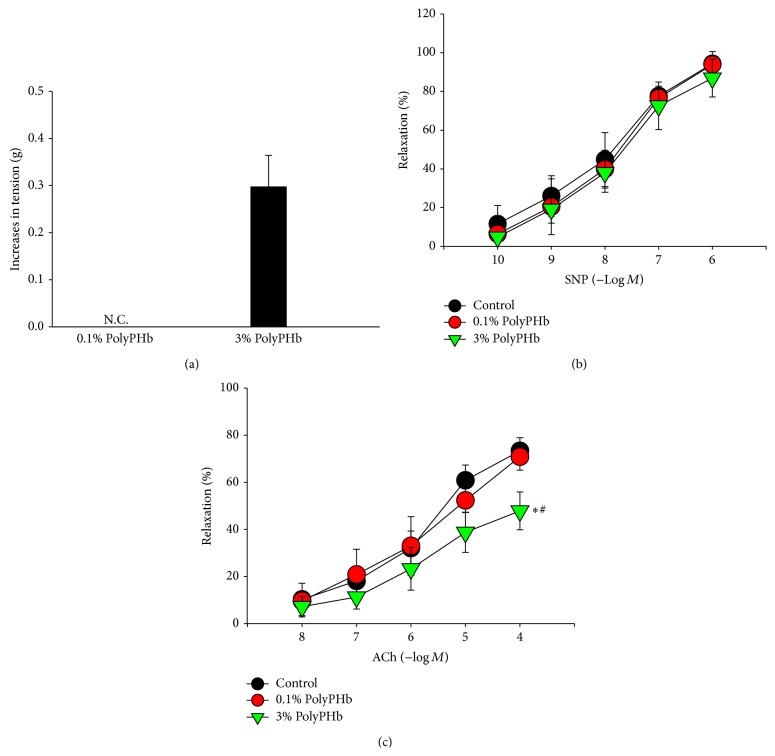
The net tension of coronary arteries (a) after incubation with 0.1% or 3% PolyPHb. SNP-induced endothelium-independent relaxation (b) and ACh-induced endothelium-dependent relaxation (c) in coronary arteries after incubation with 0.1% or 3% PolyPHb. Control vessels were treated with KH solution alone. Values are presented as mean ± SD (*n* = 5 to 6 per group). ^*^ *P* < 0.05 versus the 0.1% PolyPHb group; ^#^ *P* < 0.05 versus the Control group. SNP: sodium nitroprusside; Ach: acetylcholine; KH: Krebs-Henseleit.

Incubation with the 0.1% PolyPHb did not alter the net tension of coronary artery, whereas it was greatly elevated by the 3% PolyPHb (0.29 ± 0.07 g; Figure 5(a)). Further study found that the endothelium-independent vasorelaxation induced by SNP did not differ among groups (Figure 5(b)). However, treatment with the 3% PolyPHb induced a significant impairment in vasodilatory responses to ACh (*P* < 0.05 versus the Sham group and *P* < 0.05 versus the 0.1% PolyPHb group; Figure 5(c)).

### 3.4. High-Dose PolyPHb Induced Oxidative Stress in Coronary Artery

**Figure 6:**
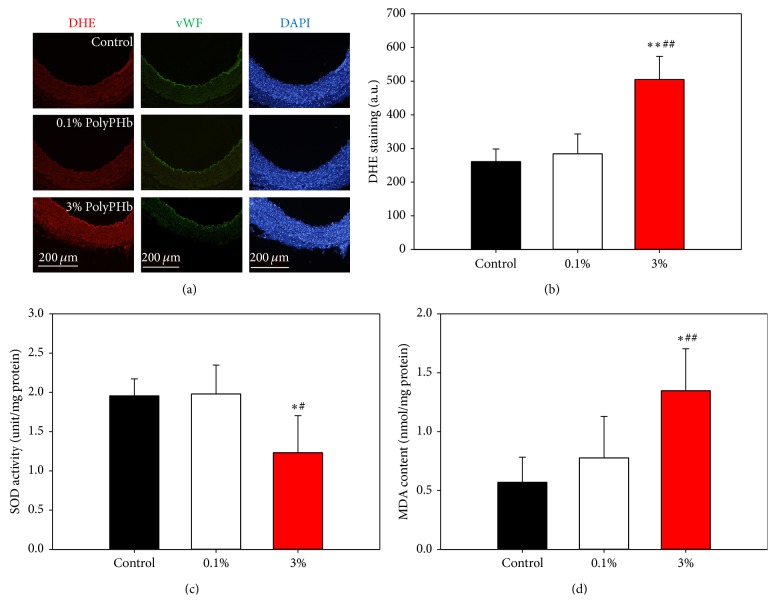
(a) Representative immunohistochemical staining of coronary arteries for DHE and vWF. Scale bar: 200 *μ*m. (b–d) The ROS production, SOD activity, and MDA formation in HUVECs after incubation with 0.1% or 3% PolyPHb. One unit of SOD activity corresponded to 50% reduction of absorbance at 550 nm. Values are presented as mean ± SD (*n* = 5 to 6 per group). ^*^ *P* < 0.05 and ^**^ *P* < 0.01 versus the 0.1% PolyPHb group; ^#^ *P* < 0.05 and ^##^ *P* < 0.01 versus the Control group. DHE: dihydroethidium; ROS: reactive oxygen species; SOD: superoxide dismutase; MDA: malonaldehyde; HUVECs: human umbilical vein endothelial cells.

An increase of positive staining of DHE was observed after coronary artery exposure to the 3% PolyPHb, indicating an overproduction of ROS in the coronary artery (Figure 6(a)). Also, the cell study confirmed that 3% PolyPHb treatment resulted in increased ROS production (*P* < 0.01 versus the 0.1% PolyPHb group; Figure 6(b)), inhibited SOD activity, and elevated MDA formation in HUVECs (*P* < 0.05 and *P* < 0.05 versus the 0.1% PolyPHb group; Figures 6(c) and 6(d)).

### 3.5. HBOC-Induced NAD(P)H Oxidase Subunit Overexpression and Activity

**Figure 7:**
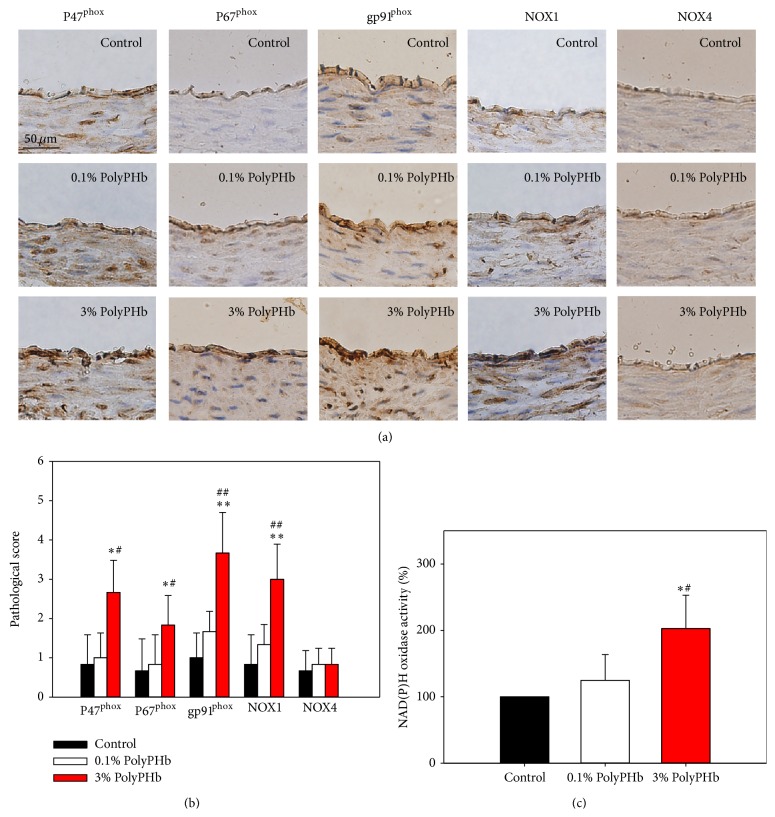
Representative of immunohistochemical staining (a) and semiquantification analysis (b) for expressions of P46^phox^, P67^phox^, gp91^phox^, Nox1, and Nox4 in coronary arteries. Scale bar: 50 *μ*m. (c) NAD(P)H oxidase activity in HUVECs. Values are presented as mean ± SD (*n* = 5 to 6 per group). ^*^ *P* < 0.05 and ^**^ *P* < 0.01 versus the 0.1% PolyPHb group; ^#^ *P* < 0.05 and ^##^ *P* < 0.01 versus the Control group.

Next, we measured the expression of the essential subunits of NAD(P)H oxidase using immunohistochemistry staining. Except for Nox4, vascular expression of p47^phox^, p67^phox^, and gp91^phox^, as well as the catalytic subunit Nox1, was markedly increased by the 3% PolyPHb, compared with control and vessels treated with the 0.1% PolyPHb (Figures 7(a) and 7(b)). Consistently, the NAD(P)H oxidase activity was also greatly upregulated by the 3% PolyPHb (*P* < 0.05 versus the Control group and *P* < 0.05 versus the 0.1% PolyPHb group; Figure 7(c)).

## 4. Discussion

As we know, in addition to cardiac I/R injury, CPB is usually companied with a reduction in hemoglobin level because of the colloid solution primed in CPB circuit and unexpected blood loss after heparinization. A higher dose of PolyPHb—3% in this study—was expected to supplement hemoglobin in circulation, thereby providing additional benefits. However, the present study provides distinct evidence that the high-dose PolyPHb cannot protect heart against CPB-induced I/R injury. With regard to some parameters, it even causes additional damage on the heart. In contrast, a clear cardioprotection is observed in the low-dose PolyPHb group, suggesting the* in vivo* cardiac effect of HBOC is highly correlated to its dosage. Moreover, our current study confirms that the high-dose PolyPHb is vasoactive and induces coronary artery endothelial dysfunction and damage. These findings to some extent may explain the paradoxical results about HBOC in animal and clinical studies. In clinical studies, a patient presenting with hypovolemic shock in hospital could receive HBOC up to 750–1000 mL, which means the estimated HBOC level in circulation is higher than 2 gHb/dL [10]. From the data of our study, this dosage is highly susceptible to induce vasoconstriction and cause damage on heart. Moreover, in the presence of physiological level of antioxidants, lower dosage of HBOC may be protective because of its peroxidase activity and excellent oxygen delivery capacity [14, 15]. However, as HBOC overwhelms the body's antioxidant defences, its own prooxidative function emerges and adverse effects become predominant.

To date, the mechanism(s) responsible for HBOC-induced vasoconstriction has not been completely understood. Scavenging of endothelium-derived nitric oxide (NO) is the most accepted theory, which proposes that the severity of vasoconstriction depends on the extent of the acellular hemoglobin extravasate through the endothelial lining of the vasculature [16]. However, there are problems with the extravasation concept. The logic of the extravasation process is not clear, because the quantities of hemoglobin molecules that can be located in the interstitium between endothelium and smooth muscle should be small compared to the blood compartment. Moreover, the amount of hemoglobin present in the interstitium would be rapidly exhausted and converted to methemoglobin (metHb), thus hindering vascular tone changes [17]. Another theory of HBOC-induced vasoconstriction is autoregulation in response to enhanced O_2_ delivery, but no direct evidence was found to support this hypothesis [18]. The data of this study suggest an “endothelial damage theory” that HBOC-induced vasoconstriction is probably due to the increased generation of ROS in the vascular endothelium and resultant endothelial dysfunction. Furthermore, we demonstrate that the high-dose PolyPHb increases the expression of NAD(P)H oxidase subunits, including P47^phox^, P67^phox^, gp91^phox^, and Nox1, suggesting that the NAD(P)H oxidase is probably responsible to HBOC-induced ROS burst and vascular redox imbalance. Although our data suggest that NAD(P)H oxidase is important to endothelial oxidative stress, we believe that this damage is multifactorial, for that excessive O_2_ delivered by HBOC and heme-auto-oxidation are both capable of producing ROS and accelerating oxidative stress. In addition, ferryl hemoglobin can mediate lipid oxidation reactions and generate powerful vasoactive molecules isoprostanes, which may also contribute to HBOC-induced vasoactivity [19].

Although HBOCs possess inherent advantages compared to stored erythrocytes, vasoactivity is regarded to be the major obstacle hindering its clinical application [17]. Reducing NO affinity has long been regarded as a solution for limitation of vasoconstriction after HBOC administration. The strategies included genetic modification of the heme pocket in hemoglobin and attenuation of HBOC extravasation through endothelial junctions by producing larger hemoglobin molecules [20, 21]. However, our study suggests that using antioxidants to counteract the oxidative damage may be a potential alternative to solve this problem. Several years ago, D'Agnillo and Change [22] reported a HBOC with antioxidant properties by cross-linking polymerized hemoglobin with SOD and catalase, which decreased the formation of oxygen radicals in a rat intestinal I/R model. Consistently, our recent study indicated that captopril, an angiotensin-converting enzyme (ACE) inhibitor with antioxidative effect, is also capable of limiting HBOC-related vasoactivity and adverse cardiac effect [23]. Therefore, manufacture of HBOC products with enhanced antioxidative properties is a possible way to reduce its vasoactivity and limit adverse cardiovascular effects.

In summary, we report that the high-dose PolyPHb fails to protect heart from CPB-induced I/R injury, which is due to induction of NAD(P)H oxidase-induced ROS overproduction and endothelial dysfunction.
