# Corrigendum to “High-Dose Polymerized Hemoglobin Fails to Alleviate Cardiac Ischemia/Reperfusion Injury due to Induction of Oxidative Damage in Coronary Artery”

**DOI:** 10.1155/2019/4576867

**Published:** 2019-03-04

**Authors:** Qian Yang, Wei Wu, Qian Li, Chan Chen, Ronghua Zhou, Yanhua Qiu, Ming Luo, Zhaoxia Tan, Shen Li, Gang Chen, Wentao Zhou, Jiaxin Liu, Chengmin Yang, Jin Liu, Tao Li

**Affiliations:** ^1^Department of Anesthesiology and Translational Neuroscience Center, West China Hospital, Sichuan University, Chengdu 610041, China; ^2^Department of Medicinal Chemistry, School of Pharmacy, Chengdu Medical College, Chengdu 610083, China; ^3^Department of Anesthesiology, Chengdu Military General Hospital, Chengdu 610083, China; ^4^Institute of Blood Transfusion, Chinese Academy of Medical Sciences, Chengdu 610052, China

In the article titled “High-Dose Polymerized Hemoglobin Fails to Alleviate Cardiac Ischemia/Reperfusion Injury due to Induction of Oxidative Damage in Coronary Artery” [1], there was an error in Figure 4(d). The authors realised when preparing another article that the representative H&E staining image for the sham group was selected in error from a similar study in rats being conducted at the same time. This image was not used for semiquantitative analysis. The corrected figure is shown below, and replicates of all the panels in Figure 4(d) are available as supplementary information.

## Figures and Tables

**Figure 1:**
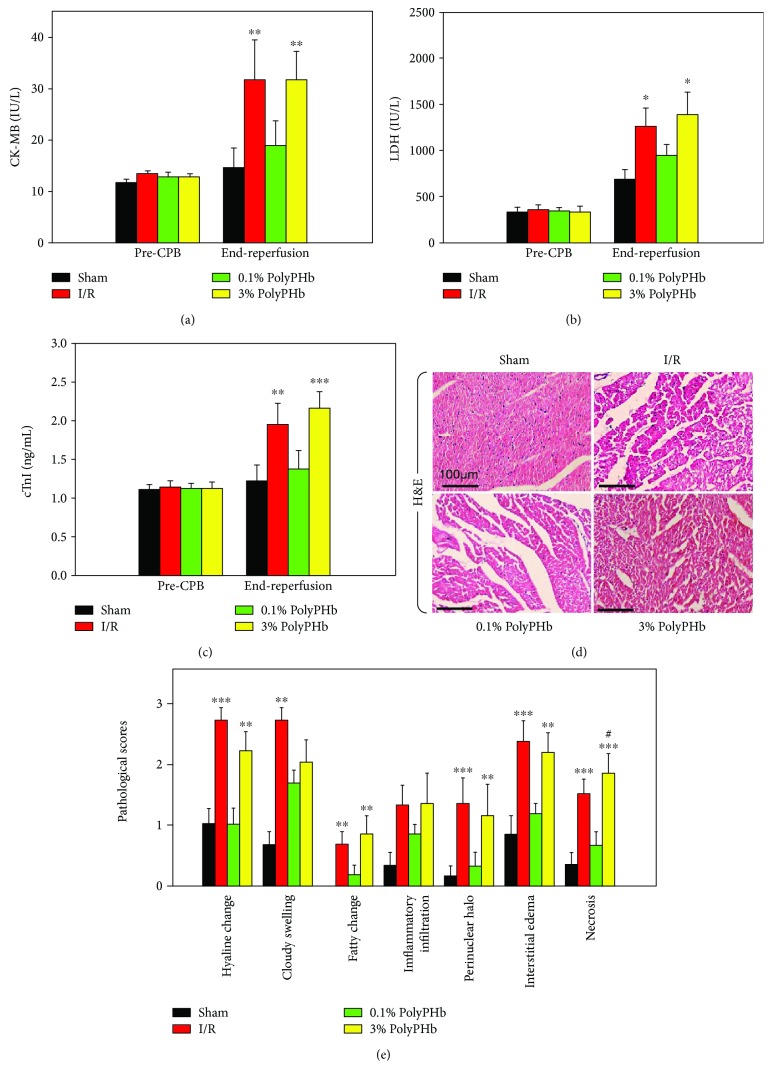
(d) The photomicrographs of H&E-stained left ventricular tissue sections from the 4 groups (*n* = 5). Scale bar: 100 *μ*m.

## Supplementary Materials

Supplementary Materials Figure 4d: the photomicrographs of H&E-stained left ventricular tissue sections from the 4 groups (*n* = 5). The pictures in the first column were selected as the representative for each group. Scale bar: 100 *μ*m.
