# Supplementary material for: Corrigendum to “High-Dose Polymerized Hemoglobin Fails to Alleviate Cardiac Ischemia/Reperfusion Injury due to Induction of Oxidative Damage in Coronary Artery”
Source: Oxid Med Cell Longev. 2019 Mar 4;2019:4576867. doi: 10.1155/2019/4576867 (PMC6425291; doi:10.1155/2019/4576867)
Supplement: Supplementary Materials — Figure 4d: the photomicrographs of H&E-stained left ventricular tissue sections from the 4 groups (n = 5). The pictures in the first column were selected as the representative for each group. Scale bar: 100 μm. [file 4576867.f1.pdf]

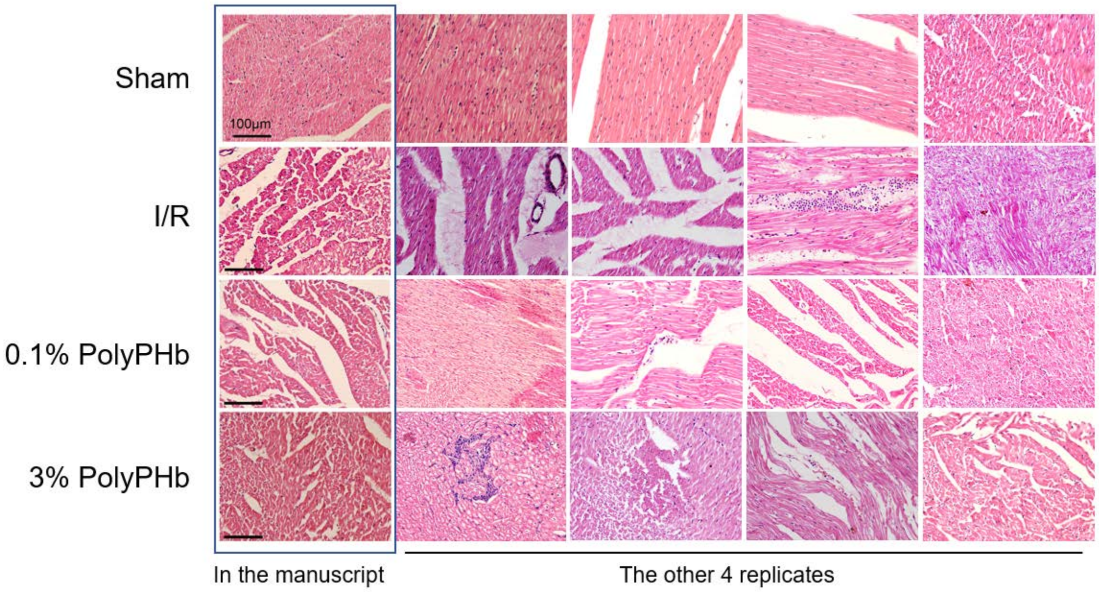

Figure 4d. The photomicrographs of H&E-stained left ventricular tissue sections from the 4 groups (n = 5). The pictures in the first column were selected as the representative for each group. Scale bar: 100 μm.
